# HIV Tropism and Decreased Risk of Breast Cancer

**DOI:** 10.1371/journal.pone.0014349

**Published:** 2010-12-16

**Authors:** Nancy A. Hessol, Laura A. Napolitano, Dawn Smith, Yolanda Lie, Alexandra Levine, Mary Young, Mardge Cohen, Howard Minkoff, Kathryn Anastos, Gypsyamber D'Souza, Ruth M. Greenblatt, James J. Goedert

**Affiliations:** 1 Department of Clinical Pharmacy, University of California San Francisco, San Francisco, California, United States of America; 2 Monogram Biosciences, South San Francisco, California, United States of America; 3 Department of Medicine, University of California San Francisco, San Francisco, California, United States of America; 4 Centers for Disease Control and Prevention, Atlanta, Georgia, United States of America; 5 City of Hope National Medical Center, Duarte, California, United States of America; 6 Georgetown University School of Medicine, Washington D.C., United States of America; 7 Departments of Medicine, Stroger Hospital and Rush University, Chicago, Illinois, United States of America; 8 Maimonides Medical Center and State University of New York, Health Sciences Center at Brooklyn, Brooklyn, New York, United States of America; 9 Montefiore Medical Center, Bronx, New York, United States of America; 10 Johns Hopkins School of Public Health, Baltimore, Maryland, United States of America; 11 Infections & Immunoepidemiology Branch, Division of Cancer Epidemiology and Genetics, National Cancer Institute, National Institutes of Health (NIH), Bethesda, Maryland, United States of America; 12 Keck School of Medicine, University of Southern California, Los Angeles, California, United States of America; INSERM, France

## Abstract

**Background:**

During the first two decades of the U.S. AIDS epidemic, and unlike some malignancies, breast cancer risk was significantly lower for women with human immunodeficiency virus (HIV) infection compared to the general population. This deficit in HIV-associated breast cancer could not be attributed to differences in survival, immune deficiency, childbearing or other breast cancer risk factors. HIV infects mononuclear immune cells by binding to the CD4 molecule and to CCR5 or CXCR4 chemokine coreceptors. Neoplastic breast cells commonly express CXCR4 but not CCR5. In vitro, binding HIV envelope protein to CXCR4 has been shown to induce apoptosis of neoplastic breast cells. Based on these observations, we hypothesized that breast cancer risk would be lower among women with CXCR4-tropic HIV infection.

**Methods and Findings:**

We conducted a breast cancer nested case-control study among women who participated in the WIHS and HERS HIV cohort studies with longitudinally collected risk factor data and plasma. Cases were HIV-infected women (mean age 46 years) who had stored plasma collected within 24 months of breast cancer diagnosis and an HIV viral load ≥500 copies/mL. Three HIV-infected control women, without breast cancer, were matched to each case based on age and plasma collection date. CXCR4-tropism was determined by a phenotypic tropism assay. Odds ratios (OR) and 95% confidence intervals (CI) for breast cancer were estimated by exact conditional logistic regression. Two (9%) of 23 breast cancer cases had CXCR4-tropic HIV, compared to 19 (28%) of 69 matched controls. Breast cancer risk was significantly and independently reduced with CXCR4 tropism (adjusted odds ratio, 0.10, 95% CI 0.002–0.84) and with menopause (adjusted odds ratio, 0.08, 95% CI 0.001–0.83). Adjustment for CD4^+^ cell count, HIV viral load, and use of antiretroviral therapy did not attenuate the association between infection with CXCR4-tropic HIV and breast cancer.

**Conclusions:**

Low breast cancer risk with HIV is specifically linked to CXCR4-using variants of HIV. These variants are thought to exclusively bind to and signal through a receptor that is commonly expressed on hyperplastic and neoplastic breast duct cells. Additional studies are needed to confirm these observations and to understand how CXCR4 might reduce breast cancer risk.

## Introduction

Human immunodeficiency virus type-1 (HIV) envelope protein binds to the CD4 receptor and to chemokine coreceptors CCR5 or CXCR4, leading to infection and destruction of the CD4-bearing immune cells: T lymphocytes and macrophages [1]. Although HIV infection increases the risk of several malignancies,[2] from 1980–2002 breast cancer risk in the United States was 31% lower among women with AIDS compared to the general population [3]. This cancer deficit was unrelated to crude measures of immune deficiency, was most pronounced before 1990, and gradually disappeared with improving antiretroviral therapy (ART) [3].

The CXCR4 receptor is commonly expressed not only on immune cells, but also on hyperplastic and especially on malignant breast duct cells [4]–[6]. CXCR4 may play an essential role in metastasis and, indirectly, earlier stages of tumor growth [4], [5], [7]–[9]. Linking HIV with breast cancer was the observation that programmed cell death (apoptosis) was induced in human breast cancer cell lines through binding of CXCR4-tropic, but not CCR5-tropic, HIV envelope protein [10]. Based on both the pattern of breast cancer risk in women with AIDS and the *in vitro* findings that CXCR4-tropic HIV induced apoptosis of breast cancer cells, we postulated that HIV strains tropic for CXCR4 may account for the reduction in breast cancer observed in HIV-infected women. To test this hypothesis, we studied HIV tropism in women with breast cancer and in matched controls.

## Methods

### Cohorts, Covariate Data and Specimens, and Ethics Statement

The study population was drawn from two large multisite longitudinal studies of HIV infection in women in the United States, the Women's Interagency HIV Study (WIHS) and the HIV Epidemiology Research Study (HERS). Study protocols were reviewed and approved by the institutional review boards, and written informed consent was obtained from the participants.

The WIHS is a prospective study of HIV infection in women, conducted in New York City, Washington D.C., Chicago, Southern California and the San Francisco Bay Area. The WIHS methods and baseline cohort characteristics have been previously described [11]. Briefly, between October 1994 and November 1995, 2056 HIV-infected and 569 uninfected women were enrolled. A second enrollment between October 2001 and September 2002, added 737 HIV-infected and 406 HIV-uninfected women [12]. Follow-up of the women enrolled in the WIHS is ongoing.

The HERS was a collaborative, multicenter (Baltimore, MD; Bronx, NY; Providence, RI; and Detroit, MI) prospective study that enrolled 871 HIV-seropositive and 439 HIV-seronegative women with acknowledged HIV risk behavior from April 1993 to January 1995. Women were enrolled on the basis of either injection drug-use or sexual risk, as has been reported previously [13]. Follow-up of the women enrolled in the HERS ended in March 2000.

The WIHS and HERS protocols for core study visits and the questionnaires used were, by design, extremely similar. At every six month core visit, women participants were interviewed, received a physical examination, and provided multiple gynecologic and blood specimens. Among HIV-infected women, blood samples collected at the core study visit were tested for CD4^+^ lymphocytes and HIV RNA load.

### Selection of Cases and Controls

Breast cancer cases were identified and confirmed through medical records and state cancer registry matches, and date of diagnosis determined. In the WIHS, cancers were identified from January 1993 through June 2009 and in the HERS from April 1993 through March 2000. Cases for the current investigation were HIV-infected women for whom we had stored plasma samples that were within 24 months (either before or after) of their cancer diagnosis and in which the HIV RNA viral load was 500 copies/mL or greater. A random sample program selected three HIV-infected control women who did not have breast cancer, had HIV RNA viral loads ≥500 copies/mL, and who matched to each case based on cohort, age (plus or minus 2 years), and date of plasma specimen collection (within six months).

### HIV Tropism Determination

The primary independent variable was HIV coreceptor usage (tropism), which was determined by the original Trofile assay (Monogram Biosciences, South San Francisco CA. Figure S1). Women were classified as having exclusively CCR5-tropic HIV (“R5”) or as having CXCR4-tropic HIV (“X4” or dual/mixed tropism “R5/X4”). The original Trofile assay has >99% sensitivity to detect low levels of CXCR4 and CCR5 variants that comprise at least 5–10% of a viral population, and the positive and negative predictive value of the assay has been verified in clinical trials of CCR5 antagonists [14].

### Statistical Analyses

All analyses were pre-specified. Contingency table analyses were conducted to compare the distribution of participant characteristics by case-control status, and chi-square or Fisher exact tests measured statistical significance. Paired t-tests were used to measure equality of means for continuous variables. Unadjusted and adjusted exact conditional, matched-pair, logistic regression was performed. The following continuous variables were transformed for the regression analyses: body mass index was divided by 10, CD4^+^ cell count was divided by 100, and HIV viral load was the log_10_. Variables that were significant at the P-value <0.10 level in the unadjusted regression models were included in the adjusted analysis. Statistical analyses were performed using SAS® software version 9.2 [15].

## Results

There were 29 confirmed breast cancer cases identified, 27 in the WIHS and 2 in the HERS. Of 29 confirmed breast cancer cases, three were excluded due to HIV viral load <500 copies/mL, and tropism results could not be determined in three others. A total of 23 breast cancer cases, who had a mean age of 46 years, were included in the analyses. Nearly all of these cancers were invasive infiltrating ductal carcinoma, and they were distributed across the years 1993–2009 (Table 1). Of 69 randomly selected controls, tropism could not be determined in seven, who were replaced with other cohort participants using the same random selection program.

**Table 1:** Histopathology and years of diagnosis of the 23 breast cancer cases in the WIHS and HERS cohort studies.

Tumor description*	Total Number
Invasive infiltrating ductal carcinoma	16
Invasive infiltrating ductal and lobular carcinoma	2
Infiltrating ductal carcinoma in situ	2
Lobular carcinoma in situ	1
Adenocarcinoma	1
Not specified	1

Year of cancer diagnosis	
1993–1996	6
1997–2000	3
2001–2004	9
2005–2008	4
2009	1

* Data obtained from medical records (pathology reports) and cancer registries (histology and behavior).

### HIV Tropism in Cases and Controls

The characteristics of the 23 cases and 69 controls with HIV tropism results are shown in Table S1. Only 2 (9%) breast cancer cases had CXCR4-tropic HIV, compared to 19 (28%) of the matched controls (Fisher's exact P = 0.09, Table S1). Two of the seven replacement controls had CXCR4-tropic HIV, for a prevalence of 29%, essentially the same as the originally selected controls. Compared to participants with CCR5-tropic HIV, those with CXCR4-tropic HIV had lower mean CD4^+^ counts (213.5 vs 389.8 cells/uL, P = 0.001) but similar mean HIV viral loads (4.4 vs 4.1 log_10_ copies/mL, P = 0.21). In addition, tropism was not associated with history of clinical AIDS or HIV viral load (P = 0.26).

### Breast Cancer Risk by HIV Tropism and Other Variables

**Figure 1:**
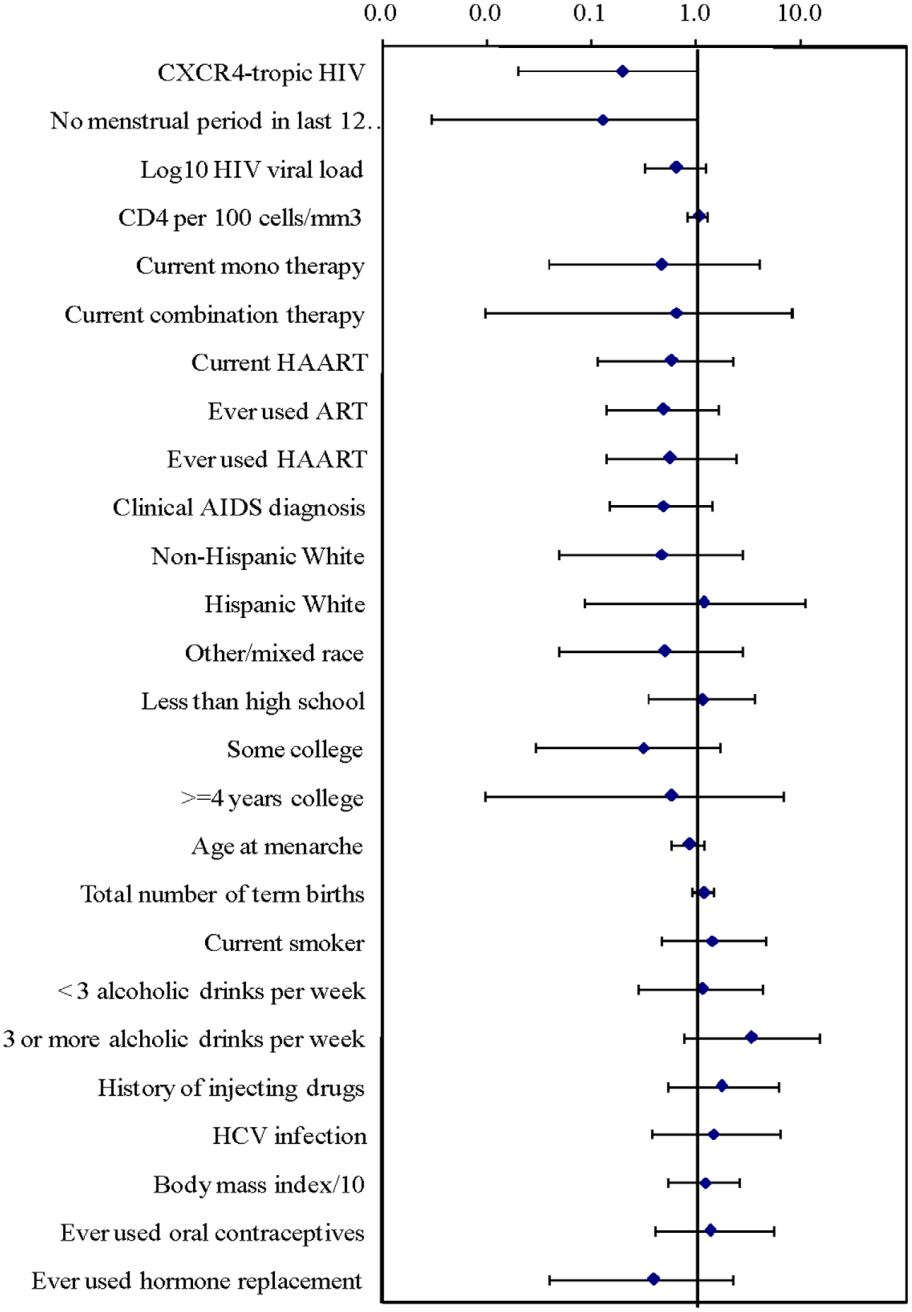
Breast cancer risk with each variable. Unadjusted conditional exact odds ratios (diamonds) and 95% confidence intervals (error bars) for breast cancer among 23 breast cancer cases and 69 controls from the WIHS and the HERS. Reference groups: no therapy (for current use of ART), African American (for race/ethnicity), high school degree (for education), and no alcohol consumption (for alcohol use).

In unadjusted exact conditional regression analysis of 20 variables (Table S1), breast cancer was marginally inversely associated with CXCR4-tropic HIV (Odds Ratio (OR) = 0.20, 95% confidence interval (CI) 0.02–1.1) as well as menopause (OR = 0.13, 95% CI 0.003–1.0), defined as not having a menstrual period for one year or more. Breast cancer was not associated with any other variables (exact P-values all >0.1), including CD4^+^ cell count, HIV viral load, ART, race/ethnicity, and classical breast cancer risk factors (Figure 1). In multivariable analysis, cancer risk was reduced in women with CXCR4-tropic HIV (adjusted OR = 0.10, 95% CI 0.002–0.84) and with menopause (adjusted OR = 0.08, 95% CI 0.001–0.83). In additional multivariable analyses, the adjusted OR and significance level for CXCR4-tropic HIV was not attenuated by the inclusion of CD4^+^ cell count, use of ART, or HIV viral load in the regression models.

## Discussion

These findings show that the odds of breast cancer in women with CXCR4-tropic HIV were 90% lower than in women with CCR5-tropic HIV. This is large enough to account for most of the breast cancer deficit for women with AIDS in the United States [3]. Our findings support the hypothesis that the low breast cancer incidence observed in women with HIV/AIDS is specifically linked to HIV variants that bind to CXCR4, a receptor that is commonly expressed on hyperplastic and neoplastic breast cells. This hypothesis was developed from three observations: 1) the utilization of CXCR4 as the coreceptor for HIV X4 and R5/X4 strains [1], 2) the common and often high level expression of CXCR4 in breast neoplasia [4]–[6], and 3) the induction of apoptosis of breast cancer cells by the specific binding of CXCR4-tropic HIV envelope to CXCR4 [10].

### CXCR4 in Breast Neoplasia

Our data showing a reduced risk of breast cancer among women with CXCR4-tropic HIV may reflect an effect of CXCR4 at an intermediate stage of breast neoplasia. Muller and colleagues first observed that CXCR4 is commonly and often highly expressed in primary breast cancers and in breast cancer cell lines [5]. This observation has been replicated and extended by many others, as reviewed by Luker and Luker [4]. Mammary stem cells express CXCR4 mRNA, as well as many other genes [16], although the relevance of this to breast neoplasia is speculative [4]. Notably, CXCR4 protein was observed by Schmid and colleagues in 13 of 14 cases of ductal carcinoma in situ of the breast, as well as in 13 of 14 areas of atypical ductal hyperplasia of the breast [6]. However, Schmid et al did not detect CXCR4 protein in normal breast epithelium [6], consistent with scanty or absent mRNA expression in cells derived from mammary epithelial tissue [5]. Overall, these data imply that normal epithelial breast cells would have few targets for, and thus probably not be directly affected by, CXCR4-tropic HIV envelope.

### CXCR4 by Cell Type

The possibility that CXCR4-tropic HIV might inhibit tumor-promoting macrophages [17] and that CXCR4 may differ between mononuclear and breast cells should be considered. CXCR4-tropic HIV envelope first binds to a CD4 epitope on T lymphocytes or macrophages and then undergoes a conformational change that allows the virus to bind to surface CXCR4 and enter the cell, causing death by various means including apoptosis [1]. CXCR4-mediated apoptosis of uninfected bystander cells that do not express CD4 has been reported, but this phenomenon commonly involves a degree of interaction with CD4-bearing cells [18]. *In vitro* experiments have reported that CD4-independent interaction of CXCR4-tropic HIV mediates apoptosis of breast cells, apparently requiring no CD4 expression or interaction to mediate conformational change of HIV and occurring without evidence of *in vitro* or *ex vivo* breast cell infection by HIV [10]. Conformational differences in the orientation or folding of surface CXCR4 [19], including conformational differences between immune versus breast cells [10], may be functionally important. The additional possibility of HIV infection of breast cells should also be considered [20].

### Assessment of Potential Survivorship, Competing Risk, and Screening Biases

Women with HIV infection may have other causes of morbidity and mortality that prevent them from being diagnosed with breast cancer or living long enough to develop breast cancer. With the increasing availability and potency of ART, these competing causes of morbidity and mortality are reduced, and thus HIV-infected women are living longer and reaching the age of higher breast cancer incidence. By matching the controls to the cases on date of plasma collection we controlled for temporal trends in the availability and potency of ART. We also matched the controls to the age of the cases. CD4^+^ cell counts were significantly lower in our participants who had CXCR4-tropic HIV. This accords with the well known tendency for patients infected with CXCR4-tropic virus to have more rapid HIV disease progression compared to those infected with only CCR5-tropic virus [1]. However, CXCR4-tropic HIV was not significantly associated with HIV viral load or history of clinical AIDS. Moreover, in multivariable models that included CD4 cell count, use of ART, or HIV viral load, the significance level for CXCR4-tropic HIV was not attenuated. As a result, we have considered and minimized competing risk as much as possible and still found a significant association between CXCR4-tropic HIV and reduced risk of breast cancer.

Screening bias may account for a fraction of the breast cancer deficit. During ages 40–49, history of screening mammography was reported by a smaller fraction of HIV-positive women in our WIHS cohort (64%) compared to the general population (79%).[21] Screening mammography history after age 50 was nearly identical in the WIHS and general populations, although the data were sparse.[21] Whether mammography screening is related to HIV tropism is unknown.

### Trends in HIV Tropism and ART Use

Initial HIV infection with HIV subtype B is almost always CCR5-tropic, with CXCR4-tropic virus emerging later, a shift that may be retarded by highly active ART (HAART) [1]. We postulate that gradual increases in HAART use and efficacy since 1996 may have been sufficient to account for the increasing trend in breast cancer incidence [3]. In 2008, 71% of HIV-infected women in the WIHS cohort were receiving HAART [N. Hessol, unpublished data]. The observed 28% prevalence of CXCR4-using HIV in our control group and 90% lower risk of breast cancer associated with these HIV strains, would account for most of the breast cancer deficit in women with AIDS [3], [22].

### Limitations and Contrary Data

We lacked *ex vivo* data to support the epidemiologic association with CXCR4-tropic HIV. In addition to the reported induction of apoptosis,[10] induction of various growth factors could contribute to or account for the association.[7]–[9] Our study was very small and limited to U.S. women who may not be representative of the global HIV epidemic. In our evaluation of classical breast cancer risk factors, we observed a lower risk associated with menopause. This association is not surprising, especially in a population with a mean age of 46 years, because early menopause is known to decrease the risk of breast cancer [23]. Despite our hypothesis-driven study that included many potential risk and confounding factors, and our exclusion of survivorship and other potential biases by matching and statistical adjustment, the association between CXCR4-tropic virus and breast cancer may be spurious due to an unmeasured viral or other exogenous or endogenous risk factor.

It should be noted that during severe immune deficiency, when CXCR4-tropic HIV is most prevalent, risks for Kaposi sarcoma and central nervous system lymphoma are very high despite tumor expression of CXCR4 [24], [25]. Nevertheless, these particular malignancies are distinct as they are known to be driven by herpes virus transformation and thus may not utilize CXCR4 as a major means of oncogenesis and metastasis. It is possible that our observed association of CXCR4-tropic HIV is unique to breast neoplasia due to conformational heterogeneity or variable surface expression of CXCR4 epitopes on neoplastic breast duct cells [10], [19].

### Summary and Implications

We found a 90% lower risk of breast cancer for women who have circulating CXCR4-tropic HIV envelope, which points to the possibility of a novel protective interaction between a specific viral protein and cancer risk. The prototype selective antagonist of CXCR4, AMD3100 (Plerixafor) [26], was reported to inhibit apoptosis of breast cancer cells by CXCR4-tropic HIV [10], and other inhibitors of CXCR4 are currently in development to treat various cancers [27]–[29]. However, derivative studies should also consider indirect pathways, such as blockade of pro-carcinogenic effects of chemokines expressed by tumor-infiltrating macrophages [17]. Continued studies of molecular interactions [8]–[10], of patients with malignancies, and of populations at risk for these diseases are needed to develop insight into the roles of CXCR4 in breast and other cancers, which may lead to new approaches for prevention or treatment.

## Supporting Information

Table S1 Characteristics of the 23 breast cancer cases and 69 controls in the WIHS and HERS cohort studies. This table provides the demographic characteristics, HIV-related parameters, and breast cancer risk factor data, as well as univariate tests of differences between cases and controls.
(0.10 MB DOC)

Figure S1 The Trofile Assay: RNA from patient plasma is subjected to RT-PCR amplification to obtain a broad representation of envelope (env) genes from HIV populations. env amplification products are then inserted into “HIV env expression vectors” (A). Patient HIV env expression vectors are co-transfected with an env-deleted “HIV genomic vector” (B) containing a firefly luciferase reporter gene that is used to quantify viral infectivity. Co-transfection of HIV env expression vectors and HIV genomic vectors produces HIV-1 pseudoviruses (C) expressing the env proteins derived from patient virus env sequences. Coreceptor tropism is determined by measuring the ability of pseudovirus populations to efficiently infect target cells co-expressing CD4 and either CXCR4 (D) or CCR5 (E) co-receptors. Co-receptor mediated infectivity is quantified by measuring luciferase infectivity in the CD4/CCR5 and CD4/CXCR4 target cells (portrayed as yellow asterisks). In the depicted example, both CXCR4+ and CCR5+ cells are infected by pseudoviruses using patient env and, therefore, viral tropism would be classified as “R5/X4” or “dual/mixed”. To confirm co-receptor usage, CCR5 and CXCR4 entry inhibitors are added to target cells (F). Viruses susceptible to CCR5 and/or CXCR4 antagonists do not produce luciferase in the corresponding target cells. env genes encoding envelope proteins capable of using the CXCR4 co-receptor, the CCR5 co-receptor, or both co-receptors are shown in green, orange and blue, respectively.
(0.27 MB PPT)
