# Supplementary material for: HIV Tropism and Decreased Risk of Breast Cancer
Source: PLoS One. 2010 Dec 16;5(12):e14349. doi: 10.1371/journal.pone.0014349 (PMC3002931; doi:10.1371/journal.pone.0014349)
Supplement: Table S1 — Characteristics of the 23 breast cancer cases and 69 controls in the WIHS and HERS cohort studies. This table provides the demographic characteristics, HIV-related parameters, and breast cancer risk factor data, as well as univariate tests of differences between cases and controls. (0.10 MB DOC) [file pone.0014349.s001.doc]

**Table S1.** Characteristics of the 23 breast cancer cases and 69 controls in the WIHS and HERS cohort studies.

|  | **Cases** | **Controls** |  |
| --- | --- | --- | --- |
| **Participant characteristic** | **N (%)** | **N (%)** | **P-value** |
| CXCR4-tropic | 2 (8.7) | 19 (27.5) | 0.09* |
| Menstrual period in the last 12 months |  |  | 0.11 |
| Yes | 17 (73.9) | 38 (55.1) |  |
| No | 6 (26.1) | 31 (44.9) |  |
| HIV RNA viral load [mean log_10_] | [3.96] | [4.21] | 0.24** |
| 500 – 4,000 copies | 6 (26.1) | 20 (29.0) | 0.83 |
| 4,000 – 49,000 copies | 11 (47.8) | 28 (40.6) |  |
| ≥50,000 copies | 6 (26.1) | 21 (30.4) |  |
| CD4+ cell count/ mm^3^ [mean] | [372] | [342] | 0.58** |
| <200 | 4 (17.4) | 23 (33.3) | 0.39* |
| 200 – 499 | 14 (60.9) | 33 (47.8) |  |
| ≥500 | 5 (21.7) | 13 (18.8) |  |
| Current HIV therapy |  |  | 0.75* |
| None | 15 (65.2) | 35 (50.7) |  |
| Mono therapy | 2 (8.7) | 10 (14.5) |  |
| Combination | 1 (4.4) | 4 (5.8) |  |
| HAART | 5 (21.7) | 20 (29.0) |  |
| Ever used ART |  |  | 0.21 |
| Yes | 15 (65.2) | 54 (78.3) |  |
| No | 8 (34.8) | 15 (21.7) |  |
| Ever used HAART |  |  | 0.55 |
| Yes | 11 (47.8) | 38 (55.1) |  |
| No | 12 (52.2) | 31 (44.9) |  |
| Self-reported clinical AIDS diagnosis |  |  | 0.19 |
| Yes | 9 (39.1) | 38 (55.1) |  |
| No | 14 (60.9) | 31 (44.9) |  |
| Age, years [mean] | [46.3] | [46.0] | 0.91 |
| 31-40 | 5 (21.7) | 21 (30.4) | 0.72 |
| 41-50 | 10 (43.5) | 26 (37.7) |  |
| > 50 | 8 (34.8) | 22 (31.9) |  |
| Race/ethnicity |  |  | 0.74* |
| White, non-Hispanic | 2 (8.7) | 12 (17.4) |  |
| White, Hispanic | 2 (8.7) | 4 (5.8) |  |
| African American | 15 (65.2) | 40 (58.0) |  |
| Other | 4 (17.4) | 13 (18.8) |  |
| Educational attainment |  |  | 0.37* |
| < High school | 10 (43.5) | 22 (31.9) |  |
| High school | 10 (43.5) | 25 (36.2) |  |
| Some college | 2 (8.7) | 17 (24.6) |  |
| ≥ 4 years college | 1 (4.3) | 5 (7.3) |  |
|  |  |  |  |
| Age at menarche [mean] | [12.6] | [13.0] | 0.43** |
| <12 | 3 (16.7) | 12 (20.3) | 0.68* |
| 12-13 | 11 (61.1) | 28 (47.5) |  |
| >13 | 4 (22.2) | 19 (32.2) |  |
| Missing | 5 | 10 |  |
| Number of term births [mean] | [3.0] | [2.3] | 0.19** |
| 0 | 4 (17.4) | 12 (17.4) | 0.54* |
| 1-2 | 8 (34.8) | 32 (46.4) |  |
| ≥3 | 11 (47.8) | 25 (36.2) |  |
| Current cigarette smoker |  |  | 0.45 |
| No | 7 (30.4) | 27 (39.1) |  |
| Yes | 16 (69.6) | 42 (60.9) |  |
| Alcohol use in past six months |  |  | 0.17* |
| None | 9 (39.1) | 36 (52.2) |  |
| <3 drinks per week | 6 (26.1) | 22 (31.9) |  |
| 3-13 drinks per week | 6 (26.1) | 10 (14.5) |  |
| ≥14 drinks per week | 2 (8.7) | 1 (1.5) |  |
| History of injection drug use |  |  | 0.27 |
| No | 11 (47.8) | 42 (60.9) |  |
| Yes | 12 (52.2) | 27 (39.1) |  |
| HCV positive at baseline |  |  | 0.38 |
| No | 8 (36.4) | 32 (47.1) |  |
| Yes | 14 (63.6) | 36 (52.9) |  |
| Missing | 1 | 1 |  |
| Body mass index |  |  | 0.98* |
| Underweight <19.8 | 2 (9.1) | 5 (7.8) |  |
| Normal 19.8-26.0 | 9 (40.9) | 30 (46.9) |  |
| Overweight 26.1-29.0 | 4 (18.2) | 11 (17.2) |  |
| Obese >29.0 | 7 (31.8) | 18 (28.1) |  |
| Missing | 1 | 5 |  |
| Ever used oral contraceptives |  |  | 0.60 |
| Yes | 17 (73.9) | 47 (68.1) |  |
| No | 6 (26.1) | 22 (31.9) |  |
| Ever used hormone replacement therapy |  |  | 0.31* |
| Yes | 2 (13.3) | 13 (29.6) |  |
| No | 13 (86.7) | 31 (70.4) |  |
| Missing | 8 | 25 |  |

* Fisher’s exact test; ** Equality of mean
