# Supplementary material for: HIV Tropism and Decreased Risk of Breast Cancer
Source: PLoS One. 2010 Dec 16;5(12):e14349. doi: 10.1371/journal.pone.0014349 (PMC3002931; doi:10.1371/journal.pone.0014349)
Supplement: Figure S1 — The Trofile Assay: RNA from patient plasma is subjected to RT-PCR amplification to obtain a broad representation of envelope (env) genes from HIV populations. env amplification products are then inserted into “HIV env expression vectors” (A). Patient HIV env expression vectors are co-transfected with an env-deleted “HIV genomic vector” (B) containing a firefly luciferase reporter gene that is used to quantify viral infectivity. Co-transfection of HIV env expression vectors and HIV genomic vectors produces HIV-1 pseudoviruses (C) expressing the env proteins derived from patient virus env sequences. Coreceptor tropism is determined by measuring the ability of pseudovirus populations to efficiently infect target cells co-expressing CD4 and either CXCR4 (D) or CCR5 (E) co-receptors. Co-receptor mediated infectivity is quantified by measuring luciferase infectivity in the CD4/CCR5 and CD4/CXCR4 target cells (portrayed as yellow asterisks). In the depicted example, both CXCR4+ and CCR5+ cells are infected by pseudoviruses using patient env and, therefore, viral tropism would be classified as “R5/X4” or “dual/mixed”. To confirm co-receptor usage, CCR5 and CXCR4 entry inhibitors are added to target cells (F). Viruses susceptible to CCR5 and/or CXCR4 antagonists do not produce luciferase in the corresponding target cells. env genes encoding envelope proteins capable of using the CXCR4 co-receptor, the CCR5 co-receptor, or both co-receptors are shown in green, orange and blue, respectively. (0.27 MB PPT) [file pone.0014349.s002.ppt]

## Slide 1
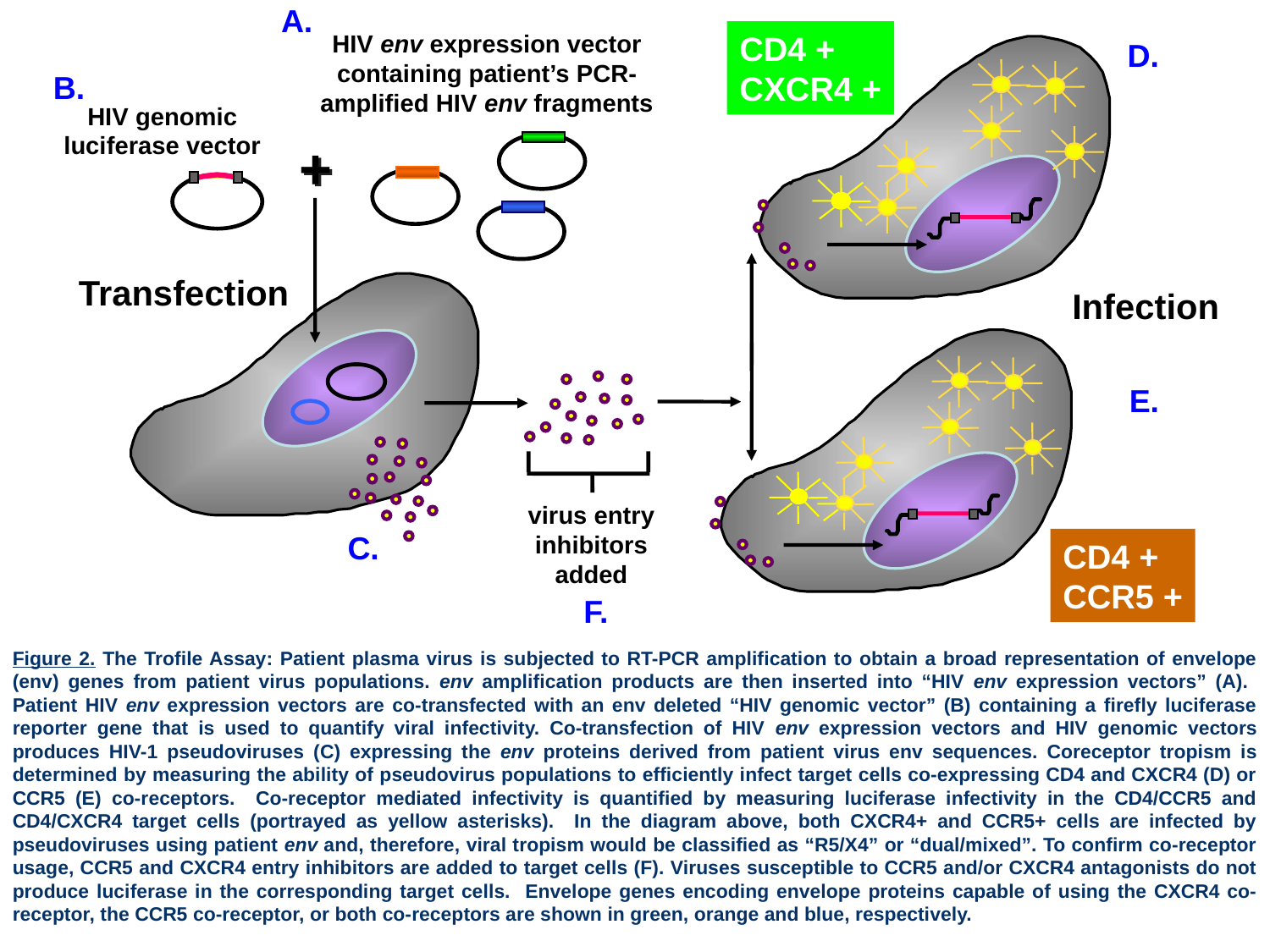

A.
CD4 +
CXCR4 +
HIV env expression vector containing patient’s PCR-amplified HIV env fragments
D.
HIV genomic
luciferase vector
+
Transfection
Infection
E.
virus entry
inhibitors
added
C.
CD4 +
CCR5 +
F.
B.
Figure 2. The Trofile Assay: Patient plasma virus is subjected to RT-PCR amplification to obtain a broad representation of envelope (env) genes from patient virus populations. env amplification products are then inserted into “HIV env expression vectors” (A). Patient HIV env expression vectors are co-transfected with an env deleted “HIV genomic vector” (B) containing a firefly luciferase reporter gene that is used to quantify viral infectivity. Co-transfection of HIV env expression vectors and HIV genomic vectors produces HIV-1 pseudoviruses (C) expressing the env proteins derived from patient virus env sequences. Coreceptor tropism is determined by measuring the ability of pseudovirus populations to efficiently infect target cells co-expressing CD4 and CXCR4 (D) or CCR5 (E) co-receptors. Co-receptor mediated infectivity is quantified by measuring luciferase infectivity in the CD4/CCR5 and CD4/CXCR4 target cells (portrayed as yellow asterisks). In the diagram above, both CXCR4+ and CCR5+ cells are infected by pseudoviruses using patient env and, therefore, viral tropism would be classified as “R5/X4” or “dual/mixed”. To confirm co-receptor usage, CCR5 and CXCR4 entry inhibitors are added to target cells (F). Viruses susceptible to CCR5 and/or CXCR4 antagonists do not produce luciferase in the corresponding target cells. Envelope genes encoding envelope proteins capable of using the CXCR4 co-receptor, the CCR5 co-receptor, or both co-receptors are shown in green, orange and blue, respectively.
